# Origins of tissue and cell-type specificity in mitochondrial DNA (mtDNA) disease

**DOI:** 10.1093/hmg/ddae059

**Published:** 2024-05-23

**Authors:** Stephen P Burr, Patrick F Chinnery

**Affiliations:** Department of Clinical Neurosciences, School of Clinical Medicine, University of Cambridge, Cambridge Biomedical Campus, Hills Road, Cambridge, CB2 0QQ, United Kingdom; Medical Research Council Mitochondrial Biology Unit, University of Cambridge, Keith Peters Building, Cambridge Biomedical Campus, Hills Road, Cambridge, CB2 0XY, United Kingdom

**Keywords:** mitochondria, single cell, heteroplasmy, mtDNA

## Abstract

Mutations of mitochondrial (mt)DNA are a major cause of morbidity and mortality in humans, accounting for approximately two thirds of diagnosed mitochondrial disease. However, despite significant advances in technology since the discovery of the first disease-causing mtDNA mutations in 1988, the comprehensive diagnosis and treatment of mtDNA disease remains challenging. This is partly due to the highly variable clinical presentation linked to tissue-specific vulnerability that determines which organs are affected. Organ involvement can vary between different mtDNA mutations, and also between patients carrying the same disease-causing variant. The clinical features frequently overlap with other non-mitochondrial diseases, both rare and common, adding to the diagnostic challenge. Building on previous findings, recent technological advances have cast further light on the mechanisms which underpin the organ vulnerability in mtDNA diseases, but our understanding is far from complete. In this review we explore the origins, current knowledge, and future directions of research in this area.

A link between defective mitochondrial function and disease was identified as early as 1959 [1], but it was a further thirty years before mutations in mtDNA began to be definitively linked to syndromes associated with mitochondrial disease, with three distinct genetic categories: rearrangements (both single large deletions or multiple deletions) of part of the mtDNA genome [2] present in a subpopulation of mtDNA molecules (heteroplasmy); single nucleotide variants (mtSNVs) present in all mtDNA molecules (homoplasmy) [3]; or heteroplasmic mtSNVs [4]. Since these initial findings, subsequent work has identified disease-causing mutations in all of the 13 protein-coding genes, 22 tRNA genes and 2 rRNA genes encoded by the mtDNA, with mutations in over 400 nuclear genes also implicated in cases of primary mitochondrial disease [5].

In the years immediately after the discovery of mtDNA disease, the most common pathogenic mtDNA mutations were quickly linked to the clinical syndromes with which they most often presented. This has resulted in the association of specific mtDNA variants with “classical” mitochondrial diseases, e.g. the heteroplasmic m.3243A>G mutation in tRNA^Leu(UUR)^ & Mitochondrial Encephalopathy with Lactic Acidosis and Stroke-like episodes (MELAS) [6]; the heteroplasmic m.8344A>G mutation in tRNA^Lys^ & Myoclonic Epilepsy with Ragged-Red Fibers (MERRF) [7]; and the homoplasmic m.11778G>A mutation in ND4 & Leber’s Hereditary Optic Neuropathy (LHON) [8]. In addition, population prevalence analysis has shown that a small number of common mutations underlie the majority of mtDNA diseases, with two studies in the northeast of England reporting that m.3243A>G and the three primary LHON mutations (m.3460G>A, m.1778G>A & m.14484T>C) account for three quarters of adult mtDNA disease diagnoses [9, 10]. However, classification and clinical management of mtDNA disease is not as simple as identifying the underlying genetic mutation and assigning a clearly defined syndrome. Multiple different mutations across the mitochondrial genome may result in the same “classical” mtDNA disease, whilst some patients carrying even the most common variants present with symptoms that do not match the disease syndrome associated with that mutation [11]. Understanding why the tissues and organs involved vary in this way is central to our understanding of the clinical presentation of these disorders but has puzzled the field for decades. Moreover, a complete explanation as to why some cell types are vulnerable—and particularly why some are resilient or protected—provides an attractive route to developing new treatments. Ultimately it is likely that multiple mechanisms contribute to the vulnerability of a given cell type to a specific mtDNA mutation (Fig. 1), and a complete understanding of these various contributing factors will be necessary to fully elucidate the complexity of mitochondrial disease.

**Figure 1:**
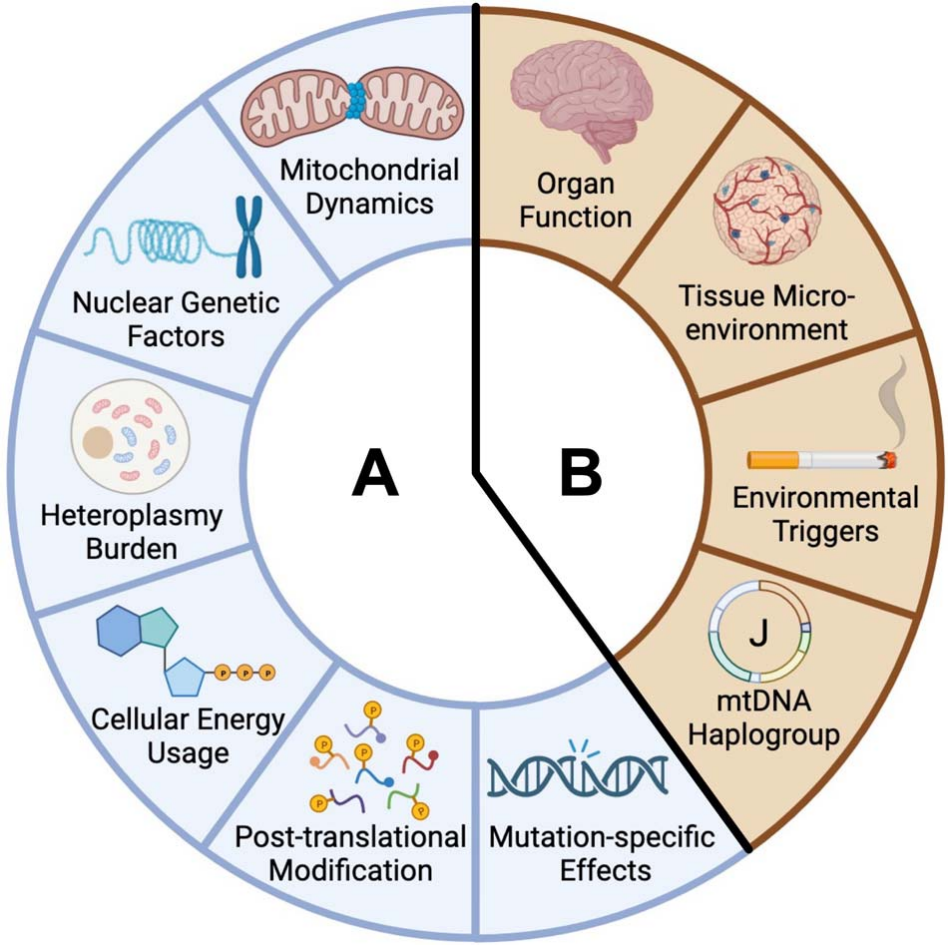
Factors affecting cell-type specificity of mtDNA disease. Multiple factors affect the susceptibility of individual cell types to mitochondrial dysfunction caused by mtDNA mutations. Many of these can vary at the single-cell level, shown in sector A, whilst others vary at the level of tissues, organs or whole organisms, shown in sector B. The combined effects of these variables dictate the exact impact of a given mtDNA mutation on an individual cell.

## Genetic bottlenecks, threshold effect & differential segregation of Heteroplasmic mtDNA mutations in humans

The majority of heteroplasmic mtDNA disease is transmitted from one generation to the next via the female germline due to the strict maternal inheritance of mtDNA seen in humans and other mammalian species [12]. In most cases, the mother carries the heteroplasmy in all oocytes, and so each offspring will inherit some level of the mutation [13]. However, some cases of mtDNA heteroplasmy arise from sporadic de-novo mutations, most often single large-scale mtDNA deletions in an individual oocyte or developing embryo, with this type of mutation very rarely being transmitted to subsequent generations [14].

### The germline genetic bottleneck

The initial mutational burden of an inherited mtDNA heteroplasmy is dictated by the ratio of mutant to wild-type (WT) mtDNA in a given oocyte. Studies in bovine and murine lineages transmitting mtDNA heteroplasmies showed that these mutations segregate rapidly between generations, with offspring from a single mother showing wide variations in the overall level of heteroplasmy inherited [15, 16]. Jenuth *et al.* hypothesised that the pattern of segregation seen in mice was due to random genetic drift during early oogenesis, with the observed rate of segregation requiring around 200 effective segregating units of mtDNA [16]. This theory, known as the germline genetic bottleneck, posits that a reduction in the number of replicating mtDNA units introduces a sampling effect in primordial germ cells (PGCs) during germline development, driving differential segregation of mutant and WT genomes [17]. Subsequent work has confirmed the presence of a germline genetic bottleneck in multiple species, including zebrafish [18] sheep [19], mice [20] and humans [21], explaining how a mother carrying heteroplasmic mtDNA mutations can give birth to children with a range of symptoms, ranging from clinically unaffected to severely ill [22]. The exact nature of the mtDNA segregating unit during the bottleneck remains a topic of debate. The simplest proposed theory suggests that mtDNA genomes segregate independently, with a rapid reduction in mtDNA copy number to around 200 copies per cell identified during PGC specification in mice [20]. However, other studies have identified either focal replication of a sub-population of mtDNAs [23] or packaging of multiple mtDNA copies into larger clusters as alternative mechanisms that define the segregating unit [24]. The role of purifying selection during germline transmission of mtDNA heteroplasmy remains an area of ongoing investigation [25] and the mechanisms involved may represent future therapeutic targets for reducing the transmission of heteroplasmic mtDNA disease.

### The biochemical and clinical threshold effects

Studies of clinical cohorts carrying many of the more common mtDNA heteroplasmies have shown that mutational burden generally correlates with the presence of clinical symptoms, with low levels of mutation found in unaffected carriers, and increasing levels of mutation resulting in progressive disease severity [26–28]. These findings support the existence of a threshold effect in heteroplasmic mtDNA disease, with functional mitochondrial defects, and subsequent clinical manifestations, only appearing when the level of mutant mtDNA exceeds a certain threshold [29]. The exact threshold heteroplasmy level can vary from mutation to mutation [30], and from tissue to tissue [31]. An important distinction can be drawn between the biochemical threshold at the cellular level, beyond which measurable mitochondrial dysfunction is seen in individual cells, and the clinical threshold in bulk tissues/organs, above which the collective effects of the mutation result in overt clinical symptoms [32]. This can be easily seen in muscle biopsies from patients with mitochondrial myopathy, which often show a mosaic pattern of unaffected and cytochrome *c* oxidase (COX)-deficient fibres when double stained for COX and succinate dehydrogenase (SDH) activity. Individual COX-deficient fibres have heteroplasmy levels above the biochemical threshold, and overall the proportion of COX-deficient vs unaffected fibres dictates the level of muscle weakness [32, 33]. Since mitochondrial defects affect the ability of cells to produce energy in the form of ATP, it is perhaps unsurprising that tissues with high energy demands, such as the central nervous system, are amongst the most commonly affected [34]. In part this could be due to the biochemical threshold in these tissues being relatively lower than other unaffected tissues. Patients carrying very high levels of mutant heteroplasmy often develop severe multisystemic disease during infancy, as the biochemical threshold is exceeded even in tissues that are less susceptible to mitochondrial dysfunction [11]. One intriguing hypothesis is that heteroplasmic mtDNA disease is driven by a lack of WT mtDNA, rather than an excess of mutant mtDNA [30] (although this may be a mutation-specific effect), and there is evidence that increased mtDNA copy number, and therefore increased WT mtDNA, may ameliorate or delay the onset of mtDNA disease in both humans [28, 35] and mice [36].

### Segregation of mtDNA Heteroplasmy

Throughout life, mtDNA is replicated and degraded continuously, independently of the nuclear DNA, in both mitotic and non-mitotic cells. Furthermore, in mitotic cells the population of mtDNAs is also split between the two daughter cells whenever cell division takes place. When heteroplasmic mtDNA mutations are present, this ongoing turnover and re-distribution of mtDNA molecules means that the initial heteroplasmy burden inherited via the maternal germline is not fixed in the offspring, but can shift over time in tissues [37]. It is well established that patients carrying the m.3243A>G MELAS mutation show an exponential decrease in heteroplasmy in blood cells with age, a phenomenon that does not occur in other tissues such as skeletal muscle [38]. Additionally, multiple case reports have identified patients carrying a range of heteroplasmy levels across different tissues [39–41]. As with germline inheritance, the dynamics of heteroplasmy segregation between and within different tissues can be governed by both random genetic drift, due to stochastic effects during mtDNA replication and/or cell division, or by active selective processes, and this remains an area of intensive study [42–44].

The combination of mechanisms influencing the mutational burden and tissue distribution of heteroplasmic mtDNA mutations goes some way to explaining why these diseases can present in such drastically different ways from patient to patient. However, other factors must be at play: for example, it has recently been reported that heteroplasmy dynamics can be influenced by the nuclear genome, adding a further layer of complexity to heteroplasmic mtDNA disease [45]. Furthermore, homoplasmic mtDNA mutations, where all tissues carry 100% mutant mtDNA, are also known to have tissue-specific effects—indicating that additional factors beyond the heteroplasmy burden must also play a role.

## Tissue specificity of Homoplasmic mtDNA disease in humans

The accumulation of homoplasmic mtDNA polymorphisms during evolution of the human mtDNA genome has resulted in the wide range of mitochondrial haplotypes present in the global population [46]. Homoplasmic mutations are maternally inherited, however, unlike heteroplasmic mutations, homoplasmic variants are present on all mtDNA molecules in all tissues of the carrier. Although a limited number of homoplasmic mutations are a common cause of mtDNA disease, there are very few definitively pathogenic homoplasmic mutations across the mitochondrial genome, particularly when compared to pathogenic heteroplasmic mtDNA mutations [33]. This suggests that most non-synonymous homoplasmic mutations are selected against during evolution. This hypothesis is backed up by studies in mouse lineages that show evidence of strong selection against transmission of deleterious homoplasmic mutations [47, 48] and analysis of human genomic databases, which reveals a predominance of synonymous and non-coding (i.e. likely non-pathogenic) homoplasmic variants in the mtDNA [49]. The most common mtDNA diseases caused by homoplasmies usually display incomplete penetrance (I.e. only some carriers are clinically affected), and often only affect a single tissue or cell-type [50], providing further evidence that more pathogenic homoplasmic variants are not compatible with human life.

By far the most common mtDNA disease is caused by homoplasmic variants is LHON [51], with rarer cases of sensorineural hearing loss (SNHL) due to RNR1 mutation [52] and maternally-inherited hypertrophic cardiomyopathy (MIHC) due to tRNA^Ile^ mutation [53] also reported. Most patients diagnosed with LHON present as adults with progressive vision loss in both eyes caused by selective loss of retinal ganglial cells (RGCs) [51], although some do present with additional neurological symptoms such as generalised dystonia [54]. LHON occurs more frequently in males (~45% of mutation carriers affected) compared to females (~10% of carriers affected) [55] and the onset of symptoms is often correlated with certain triggers, especially smoking tobacco [56]. This suggests that, in addition to the primary mtDNA mutation, genetic and environmental triggers play a critical role in the pathogenesis of LHON. Similar evidence for environmental triggering of SNHL following administration of aminoglycoside antibiotics is also well recognised [52]. Conversely it has been proposed that the risk of developing LHON may be mitigated in carriers who have increased mitochondrial biogenesis and/or higher mtDNA copy number, possibly linked to estrogen levels [57, 58], raising the possibility that external genetic and environmental factors may combine to either increase or decrease the risk of developing clinical disease on a carrier-by-carrier basis.

The incomplete penetrance and highly cell-type specific nature of LHON and other homoplasmic mtDNA diseases implies that the primary mutations themselves are only mildly deleterious, requiring additional factors to precipitate pathology in only the most susceptible cells. In addition to the well-established environmental triggers, there is some evidence to suggest that nuclear genetic modifiers may influence the penetrance of LHON, with the nuclear-encoded mitochondrial chaperone DNAJC30 [59] and PRICKLE3, involved in biogenesis of mitochondrial ATP synthase [60], being two proposed examples. Although the exact contribution of these candidate modifiers to LHON penetrance is not yet known, not least because initial findings have yet to be replicated by others, they highlight the potential importance of nuclear-mtDNA crosstalk in the dynamics of mitochondrial disease. Intriguingly, it has been hypothesised that some of the haplotype-defining variants that have accumulated during human evolution, often assumed to be non-pathogenic, are also associated with increased risk of neurodegenerative diseases [61], suggesting that classification of mtDNA homoplasmies as either pathogenic or non-pathogenic may be oversimplistic.

## Studying Tissue & Cell-type Specificity—Animal models

Much has been learned from case reports and longitudinal studies of human patient cohorts. However, detailed studies of tissue and cell-type specific aspects of mtDNA disease in humans have historically been hampered by the difficulty of obtaining samples from a sufficient range of tissues at different developmental/life stages from relatively small patient cohorts. The increasing use of high-throughput sequencing methods in recent years has begun to overcome the issue of limited patient numbers, but the challenges of obtaining clinical samples from multiple tissues throughout the lifetime of a human patient remain. The use of animal models provides a potential solution to this problem, however, generating models of mtDNA disease has proven challenging due to the absence of effective mtDNA gene editing technologies [62], although recent technological advances, discussed in detail below, promise to overcome this limitation [63]. Despite these difficulties, studies in animal models have contributed significant knowledge to the field. Whilst work in lower organisms, including *C. elegans* [64], *Drosophila* [65] & zebrafish [66] continues to provide insight into the dynamics of mtDNA segregation, mammalian species, and particularly the mouse, continue represent the best option for modelling human disease due to their comparable developmental anatomy & physiology and relative experimental tractability.

Seminal work by Shoubridge and colleagues generated one of the first heteroplasmic mouse models, fusing cytoplasts from zygotes of the NZB or BALB/c mouse strains with one-cell embryos of the other type to create a so-called “transmitochondrial” mouse, carrying a heteroplasmic mixture of NZB & BALB/c mtDNA [16]. While other groups used similar methods to generate both inter-strain [67, 68] and inter-specific [69] mtDNA mixtures, the NZB-BALB/c mouse was the first model to be studied in detail in the context of tissue-specific segregation of heteroplasmy. There are 108 nucleotide variants between NZB & BALB/c mtDNA, resulting in 15 amino acid substitutions, although these are neutral with respect to mitochondrial function in the transmitochondrial model [70, 71]. Early studies with this model identified a striking difference in the segregation pattern of the two mtDNAs between tissues as the mice aged, with blood and spleen accumulating BALB/c mtDNA, kidney and liver accumulating NZB mtDNA and other tissues including brain, muscle and skin maintaining a steady heteroplasmy [16]. Subsequent work has probed the mechanisms underlying this tissue-specific selection in liver [70] and spleen [72], with a specific role for the nuclear gene Gimap3 in leucocyte lineages [73].

Tissue-specific segregation of mtDNA heteroplasmy has subsequently been reported in other transmitochondrial strains. In studies in mice with inter-specific mtDNA heteroplasmies, the heteroplasmy segregation rate towards WT across all tissues was shown to increase as the genetic distance (I.e. number of variants) between WT and donor mtDNA genomes increased, with exact segregation patterns proving both tissue- and haplotype-specific [74]. In NZB-129 mice the pattern of selection for NZB mtDNA in liver and kidney and against in spleen matched what had been seen in NZB-BALB/c mice, although, unlike NZB-BALCB/c mice, the NZB-129 heteroplasmy proved to be deleterious, resulting in abnormal metabolism and altered behaviour in adult animals [75]. The same pattern of selection for/against NZB mtDNA was again seen in NZB-C57BL/6 mice, with evidence that segregation differs from cell-type to cell-type within tissues, rather than being uniform across individual organs, with these variations being driven by the metabolic demands of the different cell lineages [76]. A recent study utilising the NZB-C57BL/6 heteroplasmic mouse strain uncovered a role for mitophagy in differential segregation mtDNA heteroplasmy, with abolition of autophagy via *Atg7* KO resulting in neutral segregation of NZB mtDNA in liver tissue [74].

Whilst studies in transmitochondrial mice have provided important insights into the mechanisms driving tissue and cell-specific mtDNA segregation, one major drawback is that this approach does not directly model inherited mtDNA disease, which is most often caused by a single pathogenic heteroplasmic point mutation. Initial attempts to generate mice carrying such mutations focussed on using the transmitochondrial fusion technique to introduce pre-existing mtDNA mutations from somatic tissues or cell lines [48, 77]. Fan *et al.* used this technique to generate a heteroplasmic mouse model carrying a severely deleterious frameshift mutation in mt-Nd6 and a milder, yet still pathogenic, missense mutation in mt-Co1. Rapid loss of the more severe mt-Nd6 mutation was seen within four generations, whilst the mt-Co1 mutation was stably maintained in the colony over many generations, highlighting the role of purifying germline selection acting on severe mtDNA mutations [48]. However, the transmitochondrial fusion approach is severely limited by the rarity of spontaneously-occurring pathogenic mtDNA mutations in laboratory strains. Subsequently, an alternative approach to generating pathogenic mouse models employed the heterozygous mtDNA proofreading-deficient *PolgA* “mutator” mouse [78] to introduce germline mutations onto a WT mtDNA background for a single generation, before backcrossing onto WT animals to ‘isolate’ the *PolgA*-induced heteroplasmies [79]. Phenotypic screening of the resulting founder animals identified a line carrying a heteroplasmic C>T point mutation at position 5024 in the mt-tRNA^Ala^ gene, with high levels of this mutation resulting in COX-deficient colonic crypts, reduced body mass and cardiomyopathy in adult mice [79]. Furthermore, this mutation disrupts the same base-pair in the mt-tRNA^Ala^ molecule as the pathogenic human mt.5650G>A mutation, which has been reported in cases of mitochondrial myopathy [80], making this model particularly relevant in the study of mtDNA disease: Indeed, the mutation has been shown to behave similarly to pathogenic human mtDNA heteroplasmies, with a clear biochemical threshold effect, stable germline transmission and selection against the mutation in rapidly dividing tissues such as blood and colonic epithelium [79]. In addition, strong purifying selection against the m.5024C>T mutation has been noted in memory B and T lymphocytes, phenocopying the situation seen in human m.3243A>G patients [81]. Recently, a second model has been reported, produced using the same approach, that also carries a heteroplasmic variant in mt-tRNA^Ala^, this time an A>G mutation at position 5019 [82]. Again, this mutation recapitulates the behaviour of human mtDNA heteroplasmies and results in overt mitochondrial disease phenotypes in adult mice, however, biochemical analysis of the tRNA^Ala^ molecules in the two strains showed that while the mt.5024C>T mutation results in instability and reduction in steady-state levels of tRNA^Ala79^, in mt.5019A>G mice the tRNA^Ala^ molecule is stable, and it is an acylation defect that is responsible for the mitochondrial dysfunction [82]. Together these models provide an intriguing opportunity to perform comparative studies of pathogenic mtDNA heteroplasmy in the context of tissue and cell-type specific segregation.

## Single-cell & ‘omics technologies

**Figure 2:**
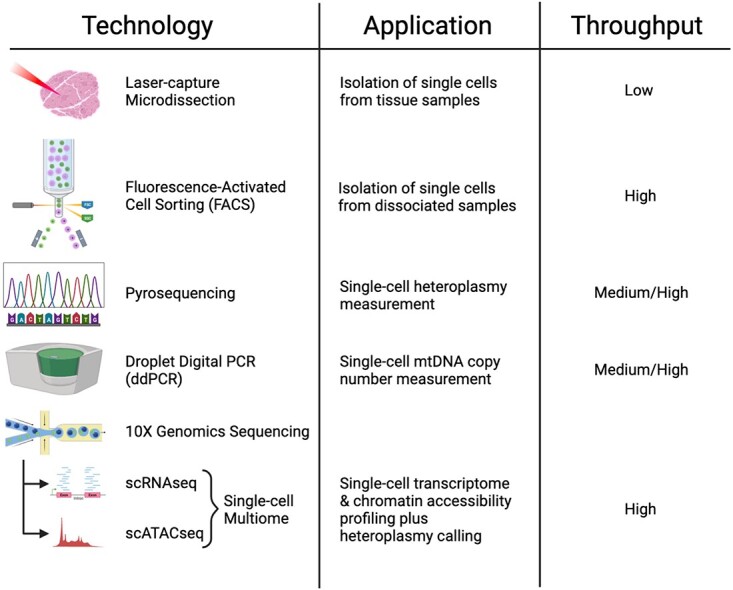
Technologies available for studying mtDNA disease in single cells. A range of techniques have been utilised to enable study of mtDNA diseases at the single-cell level, ranging from early low-throughput methods such as laser-capture microdissection of single muscle fibres through to modern high-throughput multi-modal sequencing platforms.

Historically, the study of mtDNA heteroplasmy, both in humans and animal models, has largely relied upon analysis of DNA extracted from bulk tissue samples. Whilst insights gained from these bulk tissue studies have greatly advanced our understanding of mtDNA disease, heteroplasmy can vary from cell type to cell type, and even from cell to cell. Therefore, to gain a complete understanding of these disorders, it is necessary to go beyond analysis of heterogeneous tissues and consider mtDNA dynamics at the single-cell level at scale. The importance of considering heteroplasmy at the level of individual cells has been recognised for over 30 years [83], with single-cell heteroplasmy measurements being performed on skeletal muscle fibres [4, 84], colonic crypts [36, 79] and the female germ line [20, 21] to give some examples. In this early work, cells were isolated manually or by laser capture microdissection, techniques that are generally low-throughput (Fig. 2). This made it challenging to collect enough cells to effectively investigate heteroplasmy distributions [85] and relied on comparisons between different cells based on a cellular phenotype such as a fluorescent marker [20], or histochemical staining [36]. Furthermore, subsequent analysis of the collected cells was limited to basic assays such as mtDNA heteroplasmy and mtDNA copy number measurement. However, assuming sufficient cells can be collected to allow robust statistical analysis, these approaches still uncover important new biological understanding. For example, recent work by Glynos *et al*. utilised high-throughput single-cell sorting to isolate > 5000 cells from spleen and brain tissue of m.5024C>T and m.5019A>G heteroplasmic mice at various ages ranging from embryonic day 8.5 to 1 year [84]. Single cell heteroplasmy measurements revealed progressive segregation of mtDNA heteroplasmy with age, with many cells in older m.5019A>G animals reaching mutant homoplasmy; a level of mosaicism that was not appreciated based on bulk tissue measurements alone [82] (Fig. 2).

Advances in sequencing technology have led to an explosion of single-cell methodologies, with high-throughput single-cell omics assays now commercially available (Fig. 2). This allows huge amounts of biological data to be gathered from thousands of individual cells in a single experiment with an expanding set of bespoke modifications designed to probe specific cellular functions in even finer detail [86]. Recent work utilising the m.5024C>T and m.5019A>G tRNA^Ala^ mouse models harnessed the power of high-throughput single-cell RNA sequencing (scRNAseq) to probe cell-lineage specific transcriptional changes during embryonic development by comparing ~11 000 cells from embryos carrying high levels of the two mutations to WT embryos at the same developmental stage [82]. Lineage-specific differential expression of both mtDNA transcripts and OXPHOS gene isoforms was seen in WT embryos, confirming the importance of inter-lineage mitochondrial specialisation, which is already present during early embryonic development. Furthermore, analysis of embryonic cells carrying the two tRNA^Ala^ mutations identified both lineage-specific and mutation-specific transcriptional changes, including a number of genes that appear to be involved in compensating for the mitochondrial defect caused by the heteroplasmic mutations [82]. While this study highlights the key role that single-cell technology will undoubtedly play in advancing our understanding of mtDNA disease, one potential drawback of using RNAseq alone is that it cannot directly sequence the mitochondrial genome itself. Although mtDNA sequence can be deduced from RNAseq data, the most widely used high-throughput scRNAseq platforms utilise 3′ poly-A enrichment, biasing the mtDNA coverage to the 3′ ends of poly-adenylated gene transcripts [87] and severely limiting the ability to assess heteroplasmy levels in other regions of the mtDNA genome, including tRNA genes. Currently, the modality most appropriate for investigating mtDNA heteroplasmy is the single-cell assay for transposase-accessible chromatin using sequencing (scATACseq) [88], which enriches coverage of the fully transposase-accessible mtDNA, compared to whole genome sequencing. The most common microfluidic-based scATACseq platforms require nuclei to be isolated from whole cells prior to performing the assay, a step which depletes mitochondria and poses a challenge for those wishing to sequence mtDNA at depth [89]. A modification to the standard scATACseq protocol involving permeabilization of cells, rather than complete lysis to isolate nuclei, allows for so-called mitochondrial (mt)scATACseq, effectively maintaining mtDNA within cells and significantly increasing the enrichment of mtDNA reads obtained by sequencing [90]. This technique was used to probe cell-type specific segregation of the m.3243A>G MELAS mutation in blood samples taken from patients, with heteroplasmy calling at the single-cell level identifying particularly strong selection against the mutation in T cells compared to other PBMC lineages [91].

Although individual single-cell assays, including scRNAseq and mtscATACseq, represent key technologies to investigate the dynamics of mtDNA disease, ultimately a combination of these separate, yet complementary methods will provide an even more powerful tool, allowing integrated analysis of multiple ‘omics modalities from the same sample. The introduction of high-throughput single-cell multiome analysis, coupled with a modification of the mtscATACseq method termed DOGMAseq, represents the current cutting edge of single-cell mtDNA research, allowing simultaneous profiling of transcripts, accessible chromatin, mtDNA and protein expression in individual cells [92]. Recent work by Lareau *et al.* emphasises the huge potential of these new technologies, harnessing mtscATACseq and DOGMAseq to profile over 200 000 blood cells from patients with Pearson Syndrome (PS), a disease caused by heteroplasmic single large-scale mtDNA deletions [93]. Assessment of single-cell heteroplasmy levels in cells from different immune lineages revealed strong purifying selection against the deletion in T-cells, particularly CD8 effector/memory cells and mucosal-associated invariant T-cells [93], similar to the previously mentioned findings in m.3243A>G patients [91], and in the m.5024C>T mouse model [81]. In addition, the authors utilised the vast amount of biological data generated by the combined multiomic methodologies to investigate a possible cause for the severe anaemia often associated with PS. Pseudotime analysis of the erythropoietic lineage suggested a potential role for genes in serine, glycine, and heme biosynthesis pathways as potential contributors to this phenomenon. Further advances are inevitable in this fast-moving field, and studies utilising these technologies are likely to significantly enhance our existing understanding of the origins of tissue and cell-type specificity in mtDNA disease.

## Targeted Therapeutics & Future Directions

A major long-standing challenge facing clinicians and patients is the lack of specific and targeted treatment for mtDNA disease. Whilst some mutation-specific drugs have been approved for therapeutic use, such as Idebenone, a short-chain benzoquinone effective in the treatment of LHON [94], many mtDNA diseases are treated symptomatically, with relatively few options available to target the underlying causes of the disease [95]. One potential therapeutic approach of particular interest in heteroplasmic mtDNA disease is targeted gene therapy to shift cellular heteroplasmy towards WT. To date, the two most promising methods for modulating heteroplasmy are induction of double-stranded breaks in mutant mtDNAs using mutation-specific nucleases, resulting in subsequent degradation and depletion of mutant genomes, and targeted DNA base editors that directly revert the mutant base to WT [96]. When combined with tissue-specific delivery, for example via adenoviral vectors, these technologies represent a promising prospect for future treatment of mtDNA disease, with base editors potentially able to correct both heteroplasmic and homoplasmic mutations [97, 98]. The discovery of mitochondrially-targetable DNA base editors [98] is of particular note. This new technology not only offers potential therapeutic benefits through correction of pathogenic heteroplasmies, but also enables the introduction of specific point mutations into the mtDNA genome. This promises to revolutionise research in the field, as bespoke *in vivo* and *in vitro* heteroplasmies may now be generated [63], likely leading to an exponential increase in the number of disease-relevant models becoming available in the near future.

## Conclusions: Uncertainties remain

Whilst the nature of tissue and cell-type specific heterogeneity in mtDNA disease has become increasingly clear over the past 35 years, the underlying causes of this diversity remain elusive in many cases. It seems highly likely that nuclear-mitochondrial cross-talk plays a critical role in determining cell-type vulnerability to mitochondrial defects, both at a global level, affecting fundamental processes such as mtDNA maintenance and copy number [45], and also in mutation-specific contexts, with some nuclear genes able to influence the dynamics of individual mtDNA variants [59, 60, 73]. In addition to nuclear genetic control, a number of other processes are likely also involved in modulating cell-type vulnerability to mtDNA disease, such as protein translation initiation, elongation & termination [99] and post-translational protein modifications [100], however, many aspects of these processes will ultimately be under the control of cell-type specific nuclear gene expression.

The advent of multimodal single-cell sequencing technologies has provided a tractable method to begin investigating this nuclear genetic control at the level of individual cell-lineages. Probing gene expression in two mouse models of mtDNA disease by single cell RNAseq has shown clear cell-type specific differential regulation of known genetic buffers of mitochondrial dysfunction, as well as novel transcriptional regulators that may be involved in lineage-specific compensatory mechanisms [82], highlighting the relevance of such studies in defining the origins of tissue and cell-type specificity in mtDNA disease. Whilst the unique segregation dynamics of heteroplasmic mtDNA mutations results in complex clinical presentations, the varying level of mutational burden present in these diseases may actually prove beneficial for future single-cell studies, with mtscATACseq enabling assignment of heteroplasmy levels to individual cells, thus allowing the effects of different “doses” of the mutation on the transcriptome and chromatin accessibility profiles of specific cell lineages [92, 93].

Although the tissue and cell-type specific nature of mtDNA diseases is well documented, our understanding of the mechanisms driving these differences is still far from complete. However, recent technological advances mean that we should expect to see rapid progress in this field over the coming years.

##  

*Conflict of interest statement:* None declared.

## Funding

PFC is currently funded by a Wellcome Discovery Award (226653/Z/22/Z), a Wellcome Collaborative Award (224486/Z/21/Z), the Medical Research Council Mitochondrial Biology Unit (MC_UU_00028/7), and the Biological and Biotechnology Research Council (BB/Y003209/1). This research was supported by the NIHR Cambridge Biomedical Research Centre (BRC-1215-20014). The views expressed are those of the author(s) and not necessarily those of the NIHR or the Department of Health and Social Care. Figures were created with BioRender.com.
